# Systematic Identification of Oncogenic EGFR Interaction Partners

**DOI:** 10.1016/j.jmb.2016.12.006

**Published:** 2017-01-20

**Authors:** Julia Petschnigg, Max Kotlyar, Louise Blair, Igor Jurisica, Igor Stagljar, Robin Ketteler

**Affiliations:** 1 MRC Laboratory for Molecular Cell Biology, University College London, London, WC1E 6BT, UK; 2 Princess Margaret Cancer Center, University Health Network, Toronto, M5G 2M9, Canada; 3 Department of Medical Biophysics, University of Toronto, Toronto, M5G 1L7, Canada; 4 Department of Computer Science, University of Toronto, Toronto, M5S 2E4, Canada; 5 TECHNA Institute for the Advancement of Technology for Health, Toronto, M5G 1L5, Canada; 6 Donnelly Centre, Departments of Molecular Genetics and Biochemistry, University of Toronto, Toronto, M5S 3E1, Canada; 7 Department of Molecular Genetics, University of Toronto, M5S 1A8, Canada; 8 Department of Biochemistry, University of Toronto, M5S 1A8, Canada

**Keywords:** EGF, epidermal growth factor, EGFR, epidermal growth factor receptor, ERK, extracellular signal-regulated kinase, GFP, green fluorescent protein, Grb2, growth factor receptor-bound protein 2, SH, Src homology, MAPK, mitogen-activated protein kinase, TK, tyrosine kinase, TKI, TK inhibitor, NSCLC, non-small cell lung cancer, MaMTH, mammalian membrane two-hybrid, TACC, transforming acidic coiled-coil protein, GO, gene ontology, Cub, C-terminal half of ubiquitin, TF, transcription factor, Nub, N-terminal half of ubiquitin, MOI, multiplicity of infection, EGFR-WT, EGFR-wildtype, EGFR-ex19del, EGFR-exon19 deletion, SRE, serum response element, KM, Kaplan–Meier, FGFR, fibroblast growth factor receptor, DMEM, Dulbecco's modified Eagle's medium, PEI, polyethylenimine, PBS, phosphate-buffered saline, two-hybrid screening, protein–protein interaction, EGFR, non-small cell lung cancer, oncogenic signaling

## Abstract

The epidermal growth factor receptor (EGFR) is a receptor tyrosine kinase (TK) that—once activated upon ligand binding—leads to receptor dimerization, recruitment of protein complexes, and activation of multiple signaling cascades. The EGFR is frequently overexpressed or mutated in various cancers leading to aberrant signaling and tumor growth. Hence, identification of interaction partners that bind to mutated EGFR can help identify novel targets for drug discovery.

Here, we used a systematic approach to identify novel proteins that are involved in cancerous EGFR signaling. Using a combination of high-content imaging and a mammalian membrane two-hybrid protein–protein interaction method, we identified eight novel interaction partners of EGFR, of which half strongly interacted with oncogenic, hyperactive EGFR variants. One of these, transforming acidic coiled-coil proteins (TACC) 3, stabilizes EGFR on the cell surface, which results in an increase in downstream signaling via the mitogen-activated protein kinase and AKT pathway. Depletion of TACC3 from cells using small hairpin RNA (shRNA) knockdown or small-molecule targeting reduced mitogenic signaling in non-small cell lung cancer cell lines, suggesting that targeting TACC3 has potential as a new therapeutic approach for non-small cell lung cancer.

## Highlights

• A combined screening approach involving an image-based green fluorescent protein-Grb2 translocation assay and a mammalian membrane two-hybrid protein–protein interaction assay identified 11 novel interactors of EGFR.
• Eight of those were further confirmed by co-immunoprecipitation.
• TACC3 was identified as a novel EGFR interactor, which specifically binds to oncogenic EGFR variants.
• TACC3 directly modulates EGFR stability at the cell surface and hence promotes mitogen-activated protein kinase signaling.
• Targeting TACC3 in non-small cell lung cancer cells partially resensitizes TK-resistant cells to TK inhibitors.

## Graphical Abstract

**Figure:**
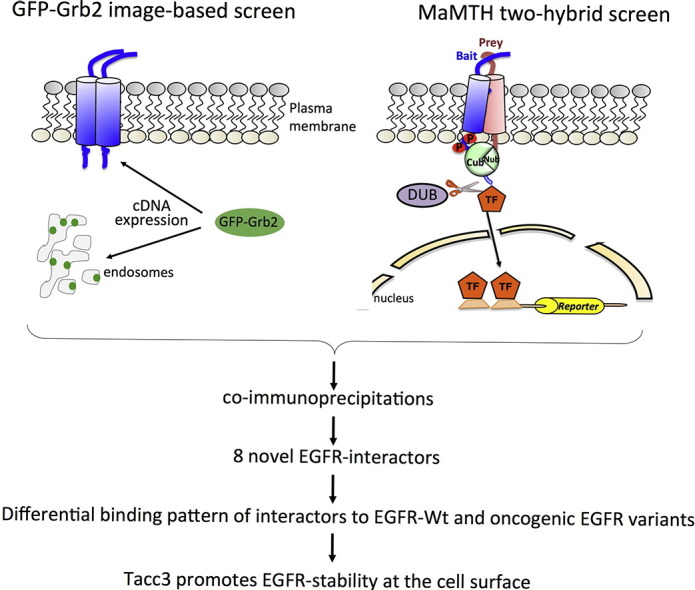

## Background

Signal transduction by growth factor receptors is essential for cells to maintain proliferation and differentiation and requires tight control. Mutations in signaling pathways frequently lead to the development of cancer [1]. Signal transduction by growth factor receptors is initiated by the binding of an external ligand to a transmembrane receptor such as the epidermal growth factor (EGF) receptor (EGFR) and activation of downstream signaling cascades [2]. A key regulator of EGFR signaling is growth factor receptor-bound protein 2 (Grb2), which is composed of an internal Src homology (SH) 2 domain flanked by two SH3 domains [3]. Grb2 binds to activated growth factor receptors at phosphorylated tyrosine residues through its SH2 domain, thus coupling receptor activation to SOS-Ras-mitogen-activated protein kinase (MAPK) signaling cascades. The modular composition of Grb2 suggests that it can dock to a variety of receptors and transduce signals along multiple pathways.

EGFR is often found overexpressed or mutated in cancer. These mutations can lead to constitutively active EGFR that triggers downstream signaling cascades, leading to uncontrolled cell growth [4]. For instance, several mutations found in lung cancer are located in the tyrosine kinase (TK) domain of EGFR. Some of these mutations, such as the L858R substitution, render the receptor susceptible to small-molecule inhibitors including the TK inhibitors (TKI) erlotinib and gefitinib [5], [6]. However, non-small cell lung cancer (NSCLC) patients who show an initial response to these drugs often develop resistance to erlotinib treatment overtime by acquiring a secondary T790M mutation [7]. Compounds such as the new FDA-approved AZD9291 (Tagrisso) aim to overcome secondary resistance mutations. However, the occurrence of tertiary mutations in these cases is a major concern. Hence, the identification of drug targets downstream of the EGFR that preferably interact with mutated EGFR receptors might overcome these problems.

While the EGFR signaling pathway is reasonably well understood, the interaction of specific signaling components with mutated EGFR is not well studied [8]. Multiple assays enable the identification of EGFR interaction partners using “OMICS” approaches [9]. However, these methods are technically challenging and costly, and until recently, genomic tools for a systematic identification of interaction partners of mutant EGFRs have been missing.

In order to systematically identify novel signaling molecules in growth factor signaling, we used a previously established microscopy-based green fluorescent protein (GFP)-Grb2 translocation assay that monitors the translocation of cytosolic GFP-tagged Grb2 to subcellular compartments upon expression of a cDNA library [10]. In a second step, to assess whether the translocation inducers participate in EGFR signaling, we tested proteins identified in the Grb2 translocation assay for their potential to bind to the EGFR using the mammalian two-hybrid (MaMTH) approach [11], which specifically allows for assaying interaction patterns of integral membrane proteins such as EGFR and allows for investigation of phosphorylation-dependent changes in interaction patterns. We found that 11 proteins bound to wild-type EGFR, and 8 of those were further confirmed by co-immunoprecipitation. Four of those eight further bound strongly to oncogenic EGFR variants that are predominantly found in NSCLC. We showed that one of them, transforming acidic coiled-coil proteins (TACC) 3, increases EGFR stability and promotes downstream EGFR signaling. Finally, we showed that targeting TACC3 in TKI-resistant NSCLC cells partially overcomes this resistance.

## Results

### Identification of novel EGFR interaction partners

**Fig. 1:**
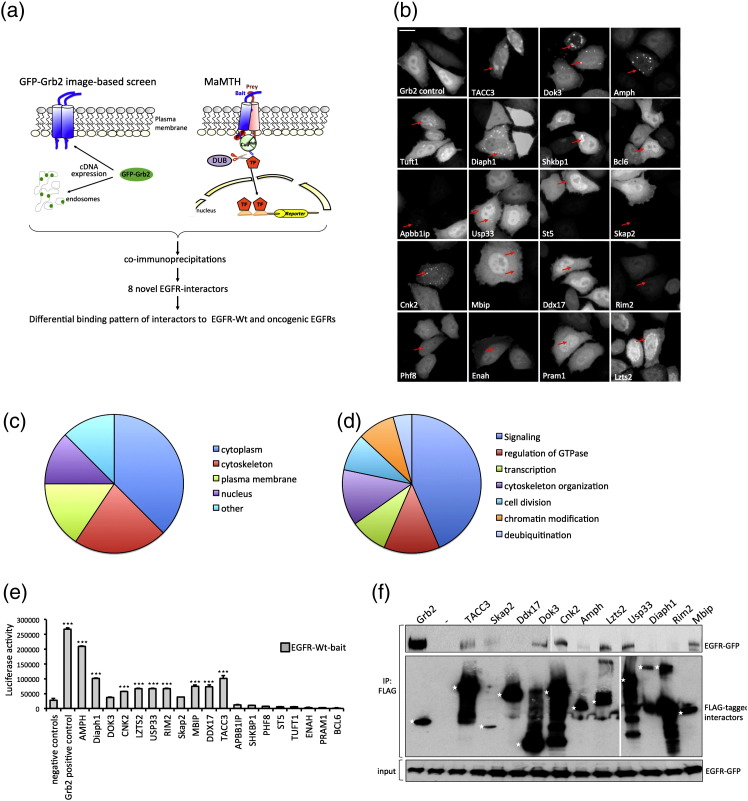
Identification of novel EGFR interaction partners. ( a) A combination of an image-based assay that monitors GFP-tagged Grb2 translocation to activated growth factor receptors and a cell-based mammalian two-hybrid assay was used to identify novel interaction partners of the EGFR. In the GFP-Grb2 translocation assay, an increase in growth factor signaling will lead to the recruitment of Grb2 to the cell surface and subsequent internalization, which is reflected by a translocation of GFP from the cytoplasm to punctate structures. In a second step, MaMTH was used to monitor the same proteins with respect to interaction with the EGFR. Briefly, an integral membrane protein (EGFR) is tagged with Cub and a TF (GAL4-NFκB). The 19 prey proteins are tagged with Nub. Both bait and prey are co-transfected or co-transduced into a stable luciferase reporter HEK293T cell line. If the bait and prey interact, ubiquitin reconstitution occurs, leading to the proteolytic cleavage by deubiquitinating enzymes (DUBs) and the subsequent release of the TF. The TF enters the nucleus, resulting in reporter gene activation. GFP-Grb2 translocation hits and MaMTH-positive interactors were further validated by co-immunoprecipitation. ( b) HeLa cells were co-transfected with GFP-Grb2 and indicated FLAG-tagged proteins, and the next day, fluorescence microscopy was performed on the PerkinElmer Opera LX high-content screening platform. Scale bar represents 20 μm. Arrows indicate areas of GFP-Grb2 recruitment. ( c) GO annotation for the localization of GFP-Grb2 translocation hits. ( d) GO annotation for the function of GFP-Grb2 translocation hits. ( e) Stable HEK293T-5xGAL4UAS luciferase reporter cells were infected with 19 indicated lentiviral preys and Grb2 as positive control, and after double stable reporter/prey cell line generation, cells were transfected with EGFR-WT. Then, 24 h after transfection, cells were lysed and luciferase activity was measured. Luciferase activity of each prey is displayed and compared to an averaged panel of negative control preys. Data are presented as means ± standard deviation (*n* = 3 biological replicates). Asterisks show *p-*values: ^⁎⁎⁎^*p* < 0.001, ^⁎⁎^*p* < 0.01, ^⁎^*p* < 0.05. ( f) HEK293T cells were co-transfected with EGFR-WT-GFP and 11 FLAG-tagged EGFR interaction partners. FLAG-Grb2 was used as positive control. The next day, cells were stimulated with EGF for 10 min and lysed. EGFR interaction partners were immunoprecipitated using anti-FLAG beads, and the association of EGFR was examined by GFP immunoblotting. Input samples were used to control for equal EGFR-WT expression. Asterisks indicate the predicted molecular weight for full-length protein of the respective interaction partner.

In order to identify novel proteins that participate in growth factor receptor signaling, we combined a previously established Grb2 translocation assay and the recently developed MaMTH system [10], [11], [12] (Fig. 1a). Given the central role of Grb2 in growth factor signaling, we designed an assay that allows the monitoring of the activation status of a cell, based on the translocation of GFP-tagged Grb2 to subcellular sites such as the plasma membrane or endosomes [10], [13]. We identified 19 proteins that induced the translocation of GFP-Grb2 from the cytosol to distinct spots upon co-transfection with GFP-Grb2 in HeLa cells (Fig. 1b). Most of the 19 proteins led to GFP-Grb2 translocation to punctate structures of variable size and number, suggesting different modes of action of the 19 translocation hits. Lzts2, TACC3, and Shkbp1 led to clusters of cytosolic GFP-Grb2, whereas Dok3, Amph, Diaph1, and Cnk2 resulted in multiple and distinct GFP-Grb2 spots. All other genes resulted in smaller punctate structures (Fig. 1b and Table 1). A punctate pattern of Grb2 localization may be indicative of Grb2 recruitment by cell surface receptors and subsequent internalization, through either direct binding or indirect activation of signaling molecules. Gene ontology (GO) annotation for localization and function for the GFP-Grb2 translocation hits showed that the majority of the 19 hits have a GO function involved in signaling pathways (43.5%) and cytoplasmic GO localization (37.5%; Table 1 and Fig. 1c and d). Some of the hits may function downstream of Grb2, but we hypothesized that some may also be linked to cell surface receptor activation. One candidate growth factor receptor that we were particularly interested in is EGFR. Therefore, in order to test whether any of these proteins are putative EGFR binding partners, we made use of the recently established MaMTH system [11], which allows for the detection of phosphorylation-dependent interactions of membrane proteins such as EGFR (Fig. 1a). Briefly, a membrane bait protein is tagged with the C-terminal half of ubiquitin (Cub) and a transcription factor (TF), and the cytosolic or membrane-bound prey is coupled to the N-terminal half of ubiquitin (Nub). Upon bait and prey interaction, the split-halves form pseudo-ubiquitin, which is recognized by cytosolic deubiqitinating enzymes, resulting in TF cleavage and reporter gene expression (Fig. 1a). For the MaMTH interaction screen, 5xGAL4UAS-luciferase reporter cells were infected with lentiviral vectors expressing the preys at a multiplicity of infection (MOI) of 0.4, selected in puromycin for 3 days, and transfected with EGFR-wildtype (EGFR-WT). Then, 24 h after transfection, cell counts were assayed and luciferase activity was measured. Our interaction score cutoff is based on the difference in luciferase activity over averaged negative controls (see Materials and Methods). Out of 19 preys, we identified 11 proteins that interacted with EGFR-WT (Fig. 1e).

To further validate the 11 novel EGFR interactors, we performed co-immunoprecipitation studies. Briefly, HEK293T cells were co-transfected with FLAG-tagged interactors and GFP-tagged EGFR-WT, and 24 h after transfection, cells were lysed and lysates were subjected to immunoprecipitation with FLAG-antibody and probed for association with EGFR by GFP immunoblotting. Moreover, 8 of 11 proteins (TACC3, Skap2, Dok3, Cnk2, Amph, Lzts2, Usp33, and Mbip) were confirmed to bind to EGFR-WT (Fig. 1f).

### Identification of novel oncogenic EGFR interaction partners

In addition to assessing the overall binding to EGFR-WT, we also tested the binding pattern of the 11 interactors to 3 mutant EGFR variants that are predominantly found in NSCLC: EGFR-L858R, EGFR-exon19 deletion (EGFR-ex19del), and EGFR-T790M/L858R. These mutant EGFR variants are constitutively active and lead to increased downstream signaling [4], [7]. L858R and ex19del render the receptor more susceptible to TKIs such as erlotinib or gefinitib, whereas the secondary T790M mutation confers resistance to TKIs [6]. We used this set of mutant EGFRs to characterize which of our novel EGFR interaction partners bind preferentially to oncogenic EGFRs using the MaMTH system.

**Fig. 2:**
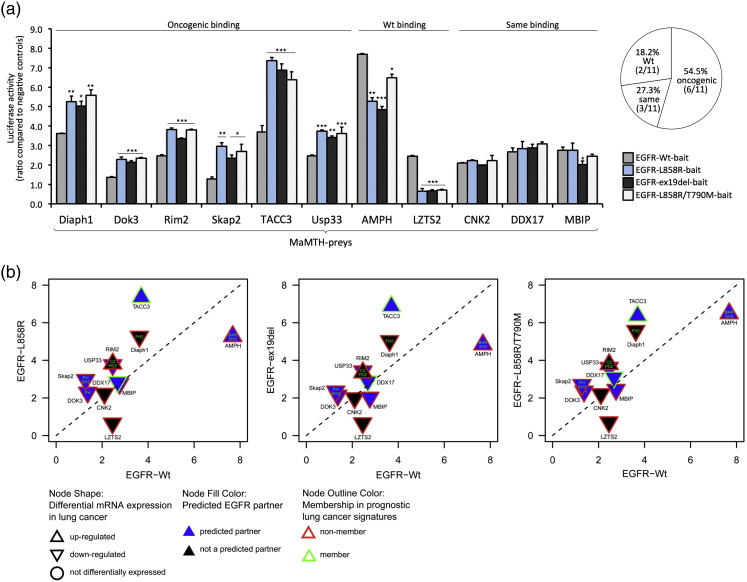
Identification of novel oncogenic EGFR interaction partners. (a) Stable HEK293T-5xGAL4UAS luciferase reporter cells were infected with 19 indicated lentiviral preys, and after double stable reporter/prey cell line generation, cells were then transfected with EGFR-WT, EGFR-L858R, EGFR-ex19del, or EGFR-T790M/L858R. Then, 24 h after transfection, cells were lysed and luciferase activity was measured. Luciferase activity is displayed as the ratio above a panel of negative control preys. Strength of interaction between each prey and EGFR-L858R, EGFR-ex19del, or EGFR-T790M/L858R is compared to the corresponding EGFR-WT interaction pattern. Data are presented as means ± standard deviation (*n* = 3 biological replicates). Asterisks show *p-*values: ^⁎⁎⁎^*p* < 0.001, ^⁎⁎^*p* < 0.01, ^⁎^*p* < 0.05. Pie chart indicates how many preys bind more strongly to EGFR-WT and the oncogenic EGFR variants or show a similar binding pattern. (b) The dot blot displays differential strength of binding to EGFR-WT compared to EGFR-L858R (left), EGFR-ex19del (middle), and EGFR-T790M/L858R (right); preys close to the *y*-axis are those that interacted more strongly with EGFR-L858R, EGFR-ex19del, or EGFR-T790M/L858R, and preys close to the *x*-axis showed stronger interaction with EGFR-WT (for MaMTH interaction scores, see Fig. 2a). Preys along the diagonal dotted line interacted similarly with WT and oncogenic mutants. Blue node fill color indicates if preys are predicted EGFR interactors. Node outline colors indicate if preys are found in prognostic lung cancer signatures, and node shape indicates if preys have been reported to be up- or down-regulated in lung cancer. Letters within the nodes indicate the presence of SH2, PTB domain, or other signaling domains.

The majority of novel interaction partners (6 out of 11, 54.5%; Diaph1, Dok3, Rim2, Skap2, TACC3, and Usp33) showed preferential binding to all three oncogenic variants compared to EGFR-WT, suggesting an increased recruitment of phosphorylation-dependent interactors. In comparison, only two preys (18.2%;Amph and Lzts2) bound preferentially to EGFR-WT and three (27.3%) showed a similar binding pattern for wild-type and mutant receptors (Cnk2, Ddx17, and Mbip; Fig. 2a).

We next sought to narrow down EGFR interactors that are most likely to be involved in EGFR signaling pathways by computational analysis and database mining. We identified seven proteins (Amph, Dok3, Ddx17, Mbip, Rim2, Skap2, TACC3, and Usp33) that are predicted to be EGFR interaction partners and/or share interaction partners within the EGFR pathway, and three of these (Amph, Rim2, and TACC3) show increased expression levels in lung cancer (Fig. 2b and Supplementary Table 1). Since several proteins involved in EGFR signaling require binding to phosphorylation sites within the cytoplasmic tail of the receptor [2], we explored whether the identified EGFR interaction partners contain any obvious EGFR binding motif such as SH2 or PTB sequences. Five proteins (Amph, Diaph1, Dok3, Rim2, and Skap2) have known signaling domains (Fig. 2b and Supplementary Table 1), but none of the 19 hits harbor SH2 or PTB sequences, (Fig. 2b and Supplementary Table 1).

We decided to investigate in detail the role of TACC3 in EGFR signaling, as it has previously not been directly linked to EGFR signaling, led to pronounced spot formation of GFP-Grb2 recruitment, and showed the strongest interaction with oncogenic EGFRs (Fig. 2a and b). Furthermore, TACC3 has previously been shown to be up-regulated in lung cancer and, based on our computational analysis, is a prognostic lung cancer marker (Fig. 2b and Supplementary Table 1).

**Fig. 3:**
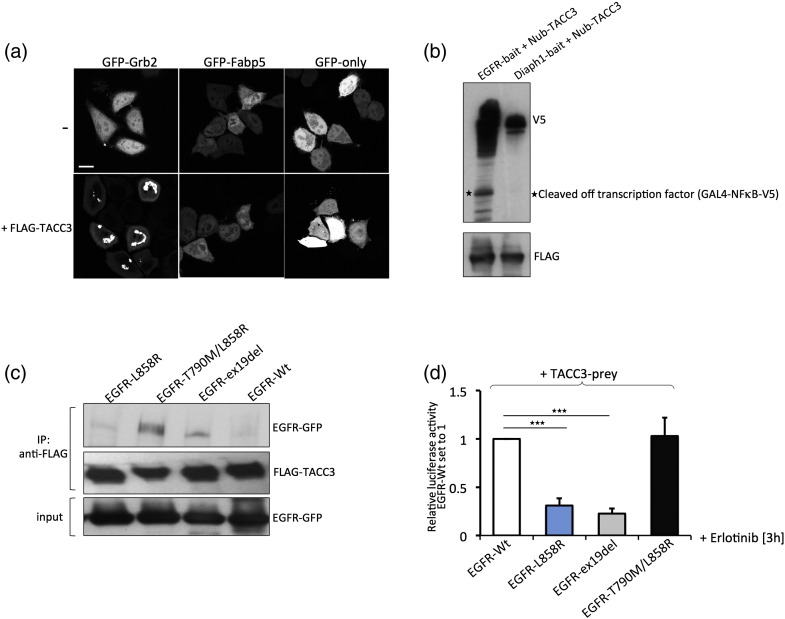
TACC3 is a novel EGFR interaction partner. (a) HeLa cells were either transfected with GFP-Grb2, GFP-only, or GFP-Fabp5 or co-transfected with FLAG-TACC3 and GFP-Grb2, GFP-only, or GFP-Fabp5. The next day, fluorescence microscopy was performed. Scale bar represents 20 μm. (b) HEK293T cells were co-transfected with EGFR-WT-bait (Cub-GAL4-NFκB-V5), Diaph1-bait, and Nub-TACC3 prey. The next day, cells were lysed and TACC3-prey expression was assessed using FLAG-antibody. Bait expression and cleaved off TF were assessed by V5 immunoblotting. (c) HEK293T cells were co-transfected with GFP-tagged EGFR-WT, EGFR-L858R, EGFR-ex19del, or EGFR-T790M/L858R and FLAG-tagged TACC3. Then, 8 h after transfection, media was changed to starvation media for additional 16 h, and cells were then lysed. TACC3 was immunoprecipitated using anti-FLAG beads, and the association of EGFR was examined by GFP immunoblotting. Input samples were used to control for equal EGFR-WT expression. (d) HEK293T cells were co-transfected with EGFR-WT, EGFR-L858R, EGFR-ex19del, or EGFR-T790M/L858R-baits and TACC3-prey. Then, 6 h after transfection, cells were starved overnight, erlotinib was added (1 μM) for 3 h, and cells were stimulated with EGF (100 ng/μl) for 10 min. Cells were lysed and luciferase assays performed. Relative luciferase activities compared to EGFR-WT are shown. Data are presented as means ± standard deviation (*n* = 3 biological replicates). Asterisks show *p-*values: ^⁎⁎⁎^*p* < 0.001.

First, in order to exclude that TACC3 leads to GFP spot formation of any GFP-tagged protein, not only GFP-Grb2, we co-expressed FLAG-TACC3 with GFP-Grb2, GFP-only, and an unrelated protein, GFP-Fabp5. Whereas TACC3 induced spot formation when co-expressed with GFP-Grb2, it did not alter cytosolic localization of both GFP-only and GFP-Fabp5, indicating that the effect of TACC3 on GFP-Grb2 translocation is specific (Fig. 3a). Next, in order to exclude that the TACC3-prey is unspecific and binds to any bait protein in our interaction assay, we performed a MaMTH assay testing the interactions between TACC3-prey and EGFR-bait, and an unrelated bait, Diaph1. Whereas TF cleavage occurred when co-expressing EGFR-bait and TACC3-prey in HeLa cells, no cleavage could be detected in HeLa cells co-expressing Diaph1-bait and TACC3-prey, suggesting the TACC3–EGFR interaction is specific. (Fig. 3b). We next tested whether TACC3 binds to oncogenic EGFR by co-immunoprecipitation. Indeed, we see an increased binding affinity of TACC3 to oncogenic EGFR variants even in the absence of growth factor or serum compared to EGFR-WT (Fig. 3c). Since oncogenic EGFR variants—unlike EGFR-WT—are phosphorylated even in starvation conditions, this suggests that TACC3 mainly binds to phosphorylated EGFR.

To investigate whether TACC3 has higher affinity for phosphorylated EGFR, we then performed MaMTH interaction assays and tested the binding of TACC3 to EGFR-WT and oncogenic EGFR variants upon erlotinib treatment in the presence of growth factors. Both L858R and exo19del mutations render EGFR more susceptible to erlotinib, which leads to rapid dephosphorylation of the mutated receptor. Briefly, stable HEK293T reporter cells were transfected with EGFR-baits and TACC3-prey, starved overnight, treated with erlotinib for 3 h, and then stimulated with epidermal growth factor (EGF) for 10 min. We could observe that while erlotinib treatment readily abolished the binding of TACC3 to EGFR-L858R and EGFR-ex19del, it did not affect the binding of TACC3 to EGFR-WT and EGFR-T790M/L858R (Fig. 3d). Thus, TACC3 preferentially binds to oncogenic EGFR variants in an erlotinib-sensitive and phosphorylation-dependent manner.

### TACC3 binds to oncogenic EGFRs and stabilizes EGFR at the cell surface

Based on our results that TACC3 binds to EGFR and preferentially to oncogenic EGFR variants, we next tested whether TACC3 can modulate EGFR-mediated signaling, either directly at the receptor level or downstream. One hypothesis was that TACC3 might stabilize EGFR on the cell surface or increase EGFR levels in general, thus keeping the receptor in a signaling-competent state for a longer period of time and/or that TACC3 modulates EGFR endocytosis.

**Fig. 4:**
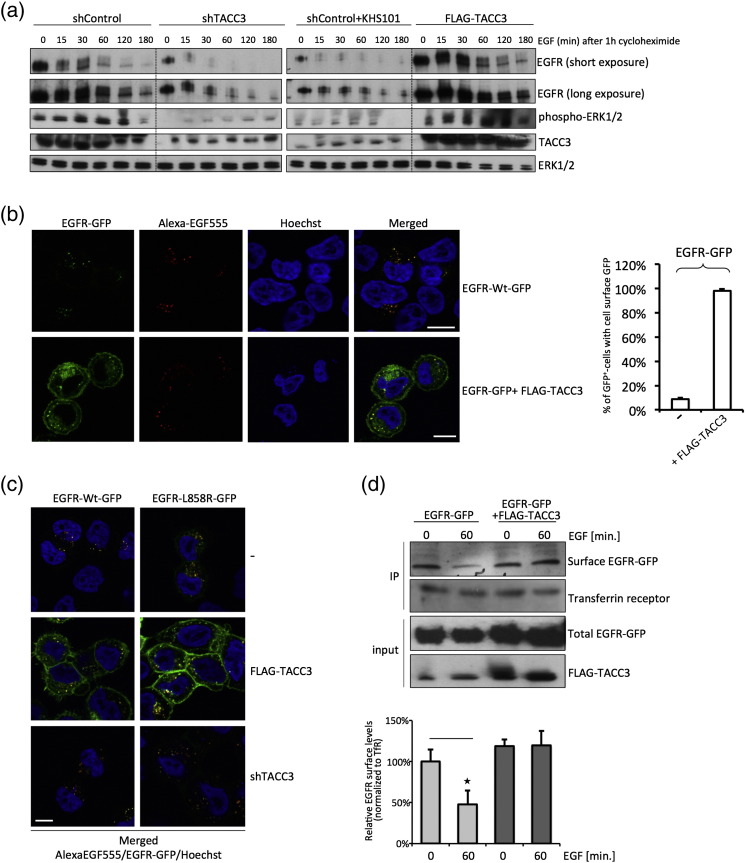
TACC3 stabilizes EGFR at the cell surface. (a) HeLa cells were seeded and transfected with indicated constructs. After 2 days, cells were starved overnight, pre-treated with KHS101 (5 μM) for 3 h (where indicated), treated with cycloheximide for 1 h, and stimulated with EGF (100 ng/μl) for indicated times to assess EGFR stability. Cells were then lysed and subjected to Western blot analysis using EGFR, phospho-ERK1/2, ERK1/2, and TACC3 antibodies. (b) HeLa cells stably expressing FLAG-TACC3 and control HeLa cells were seeded onto glass coverslips and transfected with EGFR-GFP. Then, 2 days after transfection, cells were starved overnight, then stimulated with EGF (100 ng/μl) for 60 min, and fixed;fluorescence microscopy was performed. Scale bar represents 10 μm. Then, 30 cells were counted in two independent experiments, and overall cell surface GFP was assessed. (c) HeLa cells were seeded onto glass coverslips and transfected with EGFR-GFP or EGFR-L858R-GFP and FLAG-tagged TACC3. Then, 2 days after transfection, cells were starved overnight, then stimulated with Alexa-EGF555 (100 ng/μl) for 60 min, and fixed; fluorescence microscopy was performed. Scale bar represents 10 μm. (d) HeLa cells were transfected with FLAG-tagged TACC3 or empty FLAG-vector control. Then, 2 days after transfection, cells were starved overnight and stimulated with EGF (100 ng/μl) for 60 min. Cell surface proteins were biotinylated and immunoprecipitated using streptavidin-coupled beads. Cell surface EGFR levels were assessed by Western blot analysis of immunoprecipitated samples using EGFR antibody and transferrin receptor antibody. EGFR and TACC3 expression levels of input samples were assessed. The densities of the EGFR bands were quantified using ImageJ software and normalized against transferrin receptor. Data are presented as means ± standard deviation. Asterisks show *p-*values: ^⁎^*p* < 0.05.

We first tested the effect on overall EGFR stability upon knockdown and overexpression of TACC3. We treated HeLa cells harboring either overexpressed or depleted TACC3 with cycloheximide to block protein synthesis and monitored EGFR degradation. After cycloheximide treatment, EGF-induced EGFR degradation was accelerated significantly in TACC3 knockdown cells and in cells treated with a small-molecule targeting TACC3, KHS101 [14], [15]. Interestingly, treatment with KHS101 resulted in reduced expression of TACC3 similar to shRNA-mediated knockdown, an effect that has not been reported previously. In contrast, overexpression of TACC3 led to reduced EGFR degradation compared to control cells (Fig. 4a). EGFR degradation in TACC3 knockdown cells or KHS101-treated cells started earlier when compared to mock transfected cells and was complete within 2 h. We next tested whether TACC3 directly influences cell surface EGFR levels. We transfected EGFR-GFP in HeLa cells stably expressing FLAG-TACC3, starved them overnight, and treated them with Alexa-EGF555 for 60 min. Whereas EGFR-GFP and Alexa-EGF colocalized in the cytoplasm in control cells (Fig. 4b), EGFR-GFP was mainly found at the cell surface of TACC3-overexpressing cells, with similar effects seen for EGFR-L858R (Fig. 4c); EGFR was not detectable at the plasma membrane in control (Fig. 4b and c) and TACC3 knockdown cells (Fig. 4c). Furthermore, we performed cell surface biotinylation assays, which confirmed increased cell surface EGFR expression in TACC3-overexpressing cells (Fig. 4d). In summary, we conclude that TACC3 increases EGFR stability and levels at the cell surface. However, we did not see a complete block of internalization, as evidenced by the uptake of Alexa-EGF in the cells in the presence or absence of TACC3.

### TACC3 promotes mitogenic signaling

**Fig. 5:**
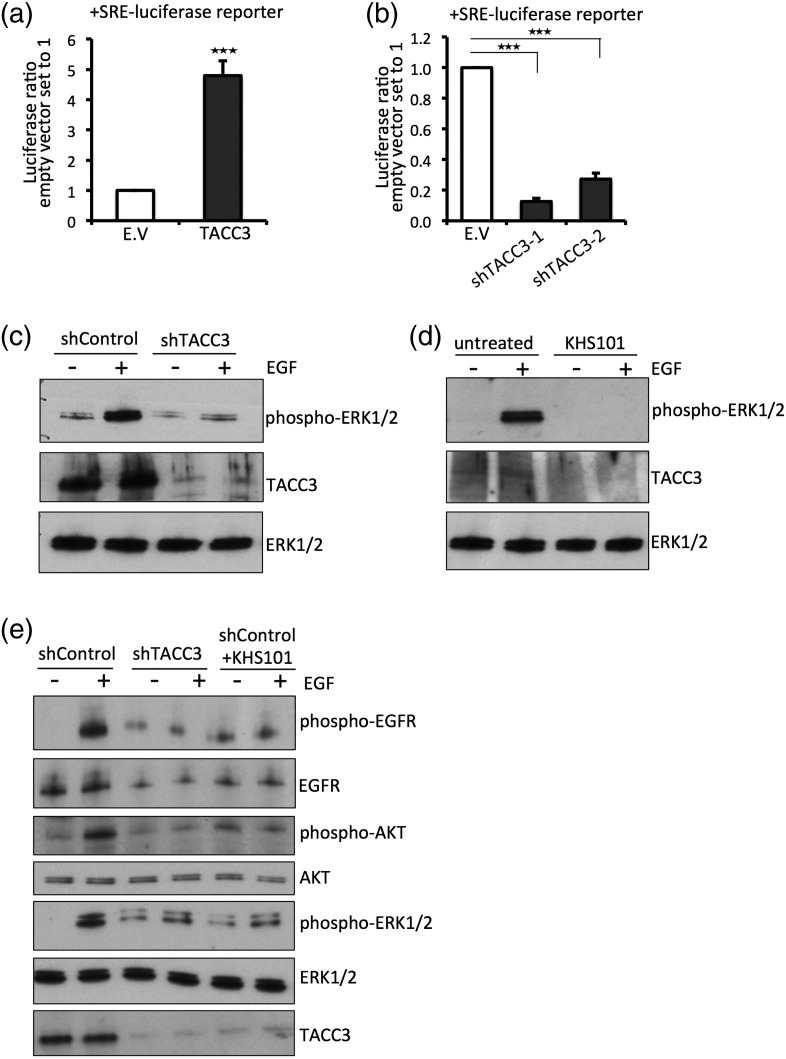
TACC3 promotes mitogenic signaling. (a) HEK293T cells were co-transfected with SRE-luciferase reporter and empty vector control (E.V.) or FLAG-tagged TACC3. Then, 3 days after transfection, cells were lysed and luciferase activity was measured. Values were normalized to the empty control vector, and changes in activity are displayed as fold values. Data are presented as means ± standard deviation (*n* = 3 biological replicates). Asterisks show *p-*values: ^⁎⁎⁎^*p* < 0.001. (b) HEK293T cells were co-transfected with SRE-luciferase reporter and empty vector control (E.V.) or two shTACC3-lentiviral constructs. Then, 3 days after transfection, cells were lysed and luciferase activity was measured. Values were normalized to the empty control vector, and changes in activity are displayed as fold values. Data are presented as means ± standard deviation (*n* = 3 biological replicates). Asterisks show *p-*values: ^⁎⁎⁎^*p* < 0.001. (c) HeLa cells were infected with indicated lentivirus (shGFP control or shTACC3) and selected for 48 h to obtain stable cells. Cells were then seeded, starved overnight, and stimulated with EGF (100 ng/μl) for 10 min. Cells were lysed and protein levels were assessed by Western blot using phospho-ERK1/2 and ERK1/2 antibodies and TACC3-antibody to control for knockdown efficiency. (d) HeLa cells were starved overnight, treated with KHS101 (5 μM) for 3 h or left untreated and then stimulated with EGF (100 ng/μl) for 10 min. Cells were lysed and protein levels were assessed by Western blot using phospho-ERK1/2, total ERK1/2 antibodies and TACC3-antibody. (e) HeLa cells were infected with indicated lentivirus (shGFP control, shTACC3) and selected for at least 48 h to obtain stable cells. Cells were then seeded, starved overnight, treated with KHS101 where indicated, and stimulated with EGF (100 ng/μl) for 10 min. Cells were lysed and protein levels were assessed by Western blot using phospho-EGFR, phospho-ERK1/2, phospho-AKT, EGFR, ERK1/2, AKT, and TACC3 antibodies.

We next tested whether TACC3 expression results in enhanced activation of mitogenic signaling pathways. First, as a readout of MAPK signaling, we used the serum response element (SRE)-luciferase luciferase reporter that is activated upon enhanced MAPK signaling via the serum response factor [16]. Briefly, HEK293T cells were co-transfected with SRE-luciferase, and luciferase activity was measured upon overexpression of TACC3 or shRNA-mediated knockdown in the presence of serum. Constitutively active K-Ras V12 was used as a positive control, and we observed a strong increase in luciferase activity upon transfection (Supplementary Fig. 1a). Upon co-transfection of TACC3 with SRE-luciferase in HEK293T cells, we observed a fivefold increase of SRE-luciferase activity upon TACC3 overexpression compared to control vector (Fig. 5a). A similar increase was also observed in starvation conditions (Supplementary Fig. 1b). Furthermore, shRNA-mediated knockdown of TACC3 in HeLa cells led to decreased SRE luciferase activity (Fig. 5b), indicating that TACC3 enhances mitogenic signaling. Next, we assayed EGFR-dependent MAPK activation through measuring the phosphorylation of extracellular signal-regulated kinase (ERK)1/2 in the presence or absence of EGF. Stimulation of HeLa cells with EGF resulted in an increase in phospho-ERK1/2 as expected, which is lost upon knockdown of TACC3 (Fig. 5c) or treatment of cells with KHS101 (Fig. 5d). Overall, these results suggest that TACC3 promotes MAPK activity downstream of EGFR signaling.

Next, we explored whether TACC3 also enhances signaling more proximal to the EGFR. Knockdown of TACC3 in HeLa cells or treatment with KHS101 resulted in a reduction of phosphorylated EGFR levels and of EGFR levels in general, when compared to mock transfected cells (Fig. 5e). As shown in Fig. 5e, knockdown of TACC3 or KHS101 treatment resulted in reduced phospho-ERK1/2 levels and reduced phospho-AKT levels. We also observed similar effects on phospho-EGFR levels in HEK293T cells (Supplementary Fig. 2). Based on our results that a decrease in downstream signaling such as phospho-ERK1/2 and phospho-AKT correlated with decreased EGFR levels (Fig. 5e), we suggest that TACC3 stabilizes EGFR at the cell surface and increases its overall expression and stability, thus increasing signaling pathways downstream of EGFR.

### Inhibition of TACC3 partially restores TKI sensitivity in TKI-resistant NSCLC cells

**Fig. 6:**
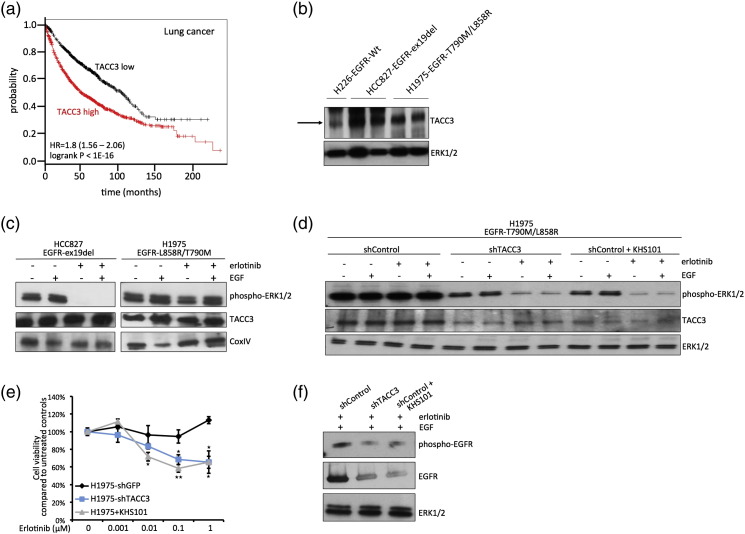
Inhibition of TACC3 partially restores TKI sensitivity in TKI-resistant NSCLC cells. (a) KM plot for the overall survival for 1926 lung cancer patients shows correlation of high TACC3 expression with poor patient survival [20]. (b) TACC3 protein levels were assessed by Western blot using cell lysates of H226-EGFR-WT, HCC827-EGFR-ex19del, and H1975-EGFR-T790M/L858R NSCLC cells and using TACC3 antibody and ERK1/2 control antibody. HCC827 and H1975 cell lysates were loaded in duplicates. (c) NSCLC cell lines HCC827-EGFR-ex19del and H1975-EGFR-T790M/L858R were seeded and starved overnight. The next day, cells were either treated with erlotinib (1 μM) for 3 h or left untreated and then stimulated with EGF (100 ng/μl) for 10 min. Cells were lysed and Western blot analysis was performed using phospho-ERK1/2, TACC3, and ERK1/2 antibodies. (d) NSCLC cell lines H1975-EGFR-T790M/L858R were infected with indicated lentivirus (shGFP control or shTACC3) and selected for 48 h. Cells were then seeded and starved overnight. The next day, cells were either treated with erlotinib (1 μM) and erlotinib plus KHS101 (5 μM) for 3 h or left untreated and then stimulated with EGF (100 ng/μl) for 10 min. Cell were lysed and Western blot analysis was performed using phospho-ERK1/2, TACC3, and ERK1/2 antibodies. (e) H1975-EGFR-T790M/L858R cells were stably infected with indicated virus (shGFP control or shTACC3), seeded after 48 h of selection, and starved overnight. The next day, cells were treated with indicated concentrations of erlotinib or erlotinib plus KHS101 overnight, and cell viability was assessed. Values are normalized to samples without erlotinib addition. Data are presented as means ± standard deviation (*n* = 3 biological replicates). Asterisks show *p*-values: **P < 0.01, *P < 0.05. (f) H1975-EGFR-T790M/L858R were stably infected with indicated lentivirus (shGFP-control or shTACC3) and selected for 48 h. Cells were then seeded and starved overnight. The next day, cells were either treated with erlotinib (1 μM) and erlotinib plus KHS101 (5 μM) for 3 h or left untreated and then stimulated with EGF (100 ng/μl) for 10 min. Cell were lysed and Western blot analysis was performed using phospho-EGFR, EGFR, and ERK1/2 antibodies.

Next, we sought to test the influence of TACC3 on EGFR signaling in NSCLC cells and tested whether the targeting of TACC3 has an effect on NSCLC cells that are resistant to TKI treatment. TACC3 has been previously shown to be overexpressed in various cancers, including lung cancer [17], [18]. Moreover, Kaplan–Meier (KM) survival analysis† [19] showed that high expression of TACC3 is significantly associated with poor survival in lung cancer patients (Fig. 6a). First, we examined TACC3 expression levels in two NSCLC cell lines that harbor hyperactive EGFR, HCC827 (ex19del) and H1975 (T790M/L858R). We show that both cell lines have increased TACC3 expression when compared to H226-NSCLC that expresses EGFR-WT (Fig. 6b).

We next tested whether targeting TACC3 has an influence on TKI sensitivity in NSCLC cells. As expected, we show that HCC827 (ex19del)-NSCLC cells are susceptible to the TKI erlotinib, as indicated by reduced phospho-ERK1/2 signaling upon erlotinib treatment, whereas H1975 cells harboring the T790M mutation are resistant to erlotinib (Fig. 6c). Interestingly, knockdown of TACC3 partly resensitized H1975 cells to erlotinib, as observed through a reduction in phospho-ERK1/2 levels (Fig. 6d) and decreased cell viability upon erlotinib addition (Fig. 6e). Similarly, treatment with the TACC3 inhibitor compound KHS101 partially resensitized H1975 cells to erlotinib (Fig. 6d and e), thus indicating that small-molecule targeting of TACC3 is a viable strategy to restore TKI sensitivity in resistant cell lines. We then tested whether the reduction in phospho-ERK1/2 is correlated with a reduction of EGFR in H1975 cells, as observed in HeLa cells (Figs. 4a and 5e). Indeed, combined targeting of TACC3 and erlotinib treatment reduced both EGFR levels and, concomitantly, phospho-EGFR levels in H1975 cells (Fig. 6f). In summary, this suggests that targeting TACC3 in NSCLC can lower overall EGFR and phospho-levels and could thus render cells more responsive to TKI treatment.

## Discussion

Abnormal EGFR signaling is the central mechanism for various cancers, often arising from mutated EGFR that triggers downstream signaling cascades, resulting in aberrant cellular growth and ultimately tumor formation [20]. EGFR signaling is complex and can diverge from one input signal, like ligand–receptor interaction, into multiple output signals, which aggravate efficient EGFR-targeted strategies. Plus, frequent resistance mutations and up-regulation of other signaling pathways pose a major hurdle to the development of clinical therapies [5], [9]. Hence, targeting proteins that associate with EGFR can increase therapeutic efficacy. Here, we identified novel EGFR interaction partners that specifically bind to oncogenic EGFR.

First, we used a high-content screening assay that monitors the distribution of GFP-Grb2 upon overexpression of a cDNA library [10]. This assay is amenable to high-throughput screening, is very cost-effective, and can relay information about localized signaling complexes in live cell or as a fixed end-point assay [13]. The Grb2 translocation assay does not discriminate between specific growth factor receptors unless a specific ligand is used. Thus, the assay can conveniently monitor the general activation state of a cell and systematically identify cellular factors that enhance general Grb2-mediated signaling. To further focus on factors that enhance EGFR signaling via Grb2, we used the recently developed protein–protein interaction detection technique MaMTH [11]. We were able to validate some GFP-Grb2 translocation hits as binding partners for the EGFR and examined the potential for interaction with oncogenic variants of the EGFR. This pipeline can be applied to other signaling receptors and pathways and will open new avenues to study other signaling-related protein-protein interaction (PPI) maps.

Using this approach, we identified and validated eight novel EGFR interaction partners, of which four bound more strongly to oncogenic EGFR. We showed that TACC3 harbors oncogenic properties, through stabilizing cell surface EGFR levels, and further showed that TACC3 can partially restore TKI sensitivity in NSCLC cells that are TKI resistant.

TACC3 is a member of transforming acidic coiled-coil proteins (TACCs), which are important players of centrosome- and microtubule-associated functions [17], [21]. TACC3 stabilizes and organizes the mitotic spindle to allow proper chromosome segregation [22]. It has been shown that Aurora A-mediated TACC3 phosphorylation is essential for its localization to mitotic spindles and centrosomes [23], [24].

Growing evidence suggests that TACC3 is found overexpressed in many human cancers, including ovarian cancer, breast cancer, squamous cell carcinoma, and lung cancer [17], [18], [25], [26]. Survival analysis using lung cancer patients (*n* = 1926) in KM Plotter shows significant association of high expression of TACC3 with poor survival (HR = 1.8; *p*-value < 1e^− 16^) [19]. Multiple roles for TACC3 in cancer progression have been described: high levels of TACC3 can lead to accumulation of DNA double-strand breaks and disrupt normal DNA damage pathways and proper chromosome segregation [27]. TACC3 has also been found as a gene fusion product with the cytoplasmic domain of the fibroblast growth factor receptor (FGFR) in glioblastoma multiforme and lung cancer [28], [29]. It has been previously shown that TACC3 is involved in cervical cancer progression and can induce epithelial–mesenchymal transition by the activation of PI3K/Akt and ERK1/2 signal transduction pathways [30]. Furthermore, it has been shown that EGF promoted TACC3 expression in an EGFR-dependent manner, supporting a link between EGFR signaling pathways and TACC3 [31]. A recent interesting study revealed that FGFR-TACC3 fusion protein can promote the resistance of EGFR-dependent cancer cell lines to EGFR/ErbB3 blockage via activation of ERK signaling. The authors showed that FGFR-TACC3 protein led to the resistance of H1975-T790M/L858R NSCLC cells to the TKI AZD9291. This study further corroborates a direct link between TACC3 and EGFR signaling [32]. Our study identified TACC3 as a novel EGFR interaction partner with multiple oncogenic properties and provides a first indication that TACC3 directly regulates EGFR signaling. TACC3 interacts preferentially with oncogenic EGFR in a phosphorylation-dependent way and promotes MAPK signaling pathways, which correlate with an increase in cell surface EGFR levels in TACC3-overexpressing cells. These results suggest that this interaction involves direct or indirect binding to phosphorylated tyrosine residues in the EGFR. However, common phospho-tyrosine binding domains are absent in TACC3. Another possibility is that interaction with the EGFR is indirect, involving the Grb2 or Shc1 adapter proteins. To date, we did not find any obvious interaction motif in TACC3 that can mediate such binding, and the molecular detail of this interaction requires further investigation. Our results suggest that TACC3 promotes overall EGFR levels and EGFR stability at the cell surface, thus increasing downstream signaling pathways that are conferred by EGFR.

We showed that targeting TACC3 through knockdown or treatment with TACC3 inhibitor KHS101 in NSCLC cells that are resistant to TKI treatment resulted in partial resensitizing of TKI-resistant NSCLC cells to the TKI erlotinib.

In summary, our study identified various, novel oncogenic EGFR interactors, and one of them, TACC3, is a potential drug target for NSCLC and could be targeted concomitantly with EGFR in order to potentiate current treatment or overcome acquired TKI resistance.

## Materials and Methods

### Plasmids and primers

Gateway-compatible entry clones: Entry clones were obtained by the human ORFeome library v5.1 or PCR amplified from Mammalian Gene Collection clones to create entry clones in pDONR223 using Gateway BP cloning technology (Invitrogen) according to the manufacturer's protocol. Entry clones were sequence verified. Expression vectors: For MaMTH interaction studies, bait and prey expression vectors from Ref. [11] (lentiviral and mammalian expression vectors) were used. All vectors used in this study are listed in Supplementary Table 2. A list of primers can be found in Supplementary Table 3.

### Lentivirus generation and stable cell line generation

Lentiviral reporter or prey plasmids were co-transfected with psPAX2 and pMD2 into HEK293T cells using X-tremeGene9 transfection reagent (Roche) and Optimem-serum-reduced media (Gibco). Then, 18 h after transfection, media were removed and replaced by viral harvesting media [Dulbecco's modified Eagle's medium (DMEM) + 1.1 g/100 mL bovine serum albumin (BSA)]. The first viral harvest was performed 24 h later, and high-BSA harvesting media were added to the cells. Again, after 24 h, the second harvest was done and combined with the first harvest, and virus was stored at − 80 °C. Lentiviral work was carried out in accordance with all Biosafety requirements.

For stable cell line generation, target cells were infected at a MOI between 0.3 and 0.5. Then, 24 h after infection, cells were selected with puromycin (2 μg/ml for HEK293T and HeLa, 1,5 μg/ml for HCC827 and H1975) for 48 h and expanded or frozen down for further assays.

### Cell culture

HEK293T and HeLa cells were cultured in DMEM with 10% fetal bovine serum and 1% antibiotics (penicillin/streptomycin). Starvation media consisted of DMEM with 1% fetal bovine serum and 1% antibiotics. Lung cancer cell lines H226, HCC827, and H1975 were maintained in RPMI-1640 supplemented with 10% fetal bovine serum and antibiotics. Starvation conditions were performed in DMEM or RPMI with 0.1% FBS. Erlotinib (Apollo Scientific Limited) was added at indicated concentrations, EGF (Sigma) was added as indicated (100 ng/μl), and KHS101 was added at 5 μM.

### Transfections

Transfection experiments were performed using polyethylenimine (PEI). Briefly, for a 12-well plate, 50 μl DMEM and PEI (10 mg/ml-stock) were added in a ratio of 4:1 to the DNA. Tubes were vortexed and incubated at room temperature for 15 min, and the DNA mixture was added dropwise to the cells. Then, 5–16 h later, the transfection mix was removed and replaced with indicated media.

### MaMTH EGFR interactor screen

Genes were cloned into lentiviral prey backbones. After lentivirus harvest, target cells (stable 5xGAL4UAS-luciferase reporter-HEK293T cells) were infected with prey viruses at a MOI of around 0.4. Cells were selected in puromycin (1.5 μg/ml) for 2 days, cell counts were performed, and equal amounts of cells were transfected with EGFR-WT, EGFR-L858R, EGFR-ex19del, and EGFR-L858R/T790M baits in triplicates. Then, 24 h after transfection, cells were lysed and luciferase assays performed. Each set of experiments contained a defined set of negative prey controls (GABBR2, PEX7, GABBR1, KCTD6, FABP5, and PEX19) in three replicates as described in Ref. [13]. Negative controls were chosen as such that there was a range of controls showing high-background luciferase activity (cytosolic proteins) and medium-background luciferase activity (membrane-bound proteins). Negative controls were averaged, and the fold change of luciferase activity was calculated for each of the preys. Three independent replicates of interaction assays were performed, and statistical significance was assessed by using one-way ANOVA.

### Cell viability assay

Cell viability assays using the Presto Blue reagent were performed according to the manual (ThermoFisher, Presto Blue Cell Viability Reagent). Briefly, 10,000 cells of indicated stable cell lines (H1975-shGFP control, H1975-shTACC3, and H1975-TACC3-OE) were seeded into 96-well plates in triplicates in a total volume of 90 μl. Then, 24 h after seeding, the media were replaced with fresh media containing indicated concentrations of erlotinib. Then, 48 h after treatment, 10 μl of Presto Blue reagent was added to each well and incubated at 37 **°**C for 10 min, and fluorescence was measured on a PerkinElmer EnVision II plate reader with the excitation/emission wavelengths set at 544/620 nm. Statistical analyses were performed using one-way ANOVA.

### SRE-luciferase assays

HEK293T cells were seeded at 40,000 cells/well in a 24-well dish. The next day, TACC3 overexpression constructs or lentiviral shTACC3 constructs were transfected together with either SRE-luciferase or Atg4-luciferase constructs. Then, 3 days after transfection, cells were lysed in RIPA buffer, 10 μl was transferred into 96-well plates, and 100 μl of BrigthGlo (Promega) luciferase substrate was added. Luminescence was measured on a PerkinElmer EnVision II plate reader. Luciferase activity was measured in triplicates and compared to empty vector control. Statistical analyses were performed using one-way ANOVA.

### Antibodies and Western blot analysis

Western blot analysis was performed using standard protocols. Briefly, cells were lysed in RIPA buffer containing phosphatase inhibitor (PhosSTOP, Roche) and protease inhibitor (EDTA-free tablets, Roche) on ice for 10 min, and 2x sample buffer was added, and samples were immediately boiled at 95 °C for 5 min and proceeded for Western Blot analysis. Then, 5–10% of the lysates were separated by 10% SDS-PAGE gels and transferred to nitrocellulose membranes (BioRad). Transferred samples were immunoblotted with primary antibodies, followed by incubation with HRP-coupled secondary antibodies, and detection was performed using EZ-ECL enhanced luminescence detection kit (Biological Industries). A list of antibodies used can be found in Supplementary Table 4.

### Co-immunoprecipitations

HEK293T cells were seeded at 300,000 cells/well in a 6-well dish. The following day, cells were co-transfected with plasmids encoding Flag-tagged candidate EGFR interactors and GFP-tagged EGFR. After 24-h transfection, cells were stimulated with EGF (100 ng/ml) for 5 min, washed twice with ice-cold phosphate-buffered saline (PBS), and lysed in 500 μl NP40 lysis buffer [50 mM Hepes-NaOH (pH 8), 100 mM NaCl, 1 mM EGTA, 0.5% NP40, 2.5 mM MgCl_2_, 1 mM DTT, and 10% glycerol] supplemented with protease and phosphatase inhibitors (PhosSTOP tablets from Roche; Complete EDTA-free protease inhibitor tablets from Roche). The total cell lysates were centrifuged at 20,800*g* for 30 min, and 20 μl supernatant was immediately frozen (= input samples). Flag-tagged candidate proteins were immunoprecipitated by incubating lysates with 25 μl anti-Flag M2 antibody-conjugated agarose (50% slurry) for 3–4 h at 4 °C. Beads were washed three times with lysis buffer, and proteins were eluted by adding 2x sample buffer (SB) plus β-mercaptoethanol. Proteins were resolved on 10% SDS-PAGE. Levels of GFP-EGFR were detected by immunoblotting using anti-GFP antibodies.

### Fluorescence microscopy

EGFR-GFP and FLAG-tagged TACC3 were transfected into HeLa cells seeded on glass slips. Cells were starved overnight and treated with EGF (100 ng/μl) for indicated times, and 24 h after transfection, cells were fixed in 4% paraformaldehyde and stained with Hoechst33342, and fluorescence microscopy was performed. Cells were examined using a Leica TCS SPE confocal microscope (SPE3) with GFP and Texas Red filters.

For Alexa-EGF555 endocytosis assays, cells were transfected with indicated constructs and starved overnight; Alexa-EGF555 was added (50 ng/μl) and cells were kept on ice for 30 min before being released into 37 °C incubator for indicated times. Cells were then washed twice with ice-cold PBS, fixed in 4% PFA, and stained with Hoechst33342, and fluorescence microscopy was performed.

### GFP-Grb2 translocation assay

HeLa cells were seeded in 96-well plates (Perkin Elmer, ViewPlate-96 Black, Optically Clear Bottom) and were transfected (PEI-transfection) with 100 ng GFP-Grb2 and 100 ng 19 FLAG-tagged interactors 24 h after seeding. The next day, cells were fixed with 4% paraformaldehyde and stained with Hoechst33342, and imaging was performed using the Perkin Elmer Opera LX high-content screening confocal microscope using a 40 × objective.

### EGFR predictions and bioinformatics analysis

Known and predicted interaction partners of EGFR were downloaded from the Integrated Interactions Database [33] version 2015-09. Protein domains were retrieved from the UniProt database [34] release-2015_08. Lung cancer prognostic signatures and differential gene expression data were downloaded from the LCDIP database (D. Strumpf, unpublished results), which includes prognostic signatures from Ref. [35] and other sources.

Prognostic properties of TACC3 were evaluated using http://kmplot.com (version 2015; data downloaded on March 6, 2016) [19]. Both adeno and squamous cell lung cancer samples were used, and biased samples were removed (*n* = 1926). Probe 218308_at, auto select best cutoff and censor at threshold value was used. Obtained hazard ratios and corresponding *p*-values were plotted. Resulting KM plots for overall survival are included in Fig. 6a.

### Cell surface biotinylation assay

Cells were starved overnight, treated with EGF (100 ng/μl) for indicated times, and washed twice with ice-cold PBS. Cells were incubated for 15 min on ice with 0.5 mg/ml EZ Link™ Sulfo-NHS-SS-Biotin (Pierce) and were washed twice with 100 mM glycine/PBS to quench the reaction. Then, 500 μl lysis buffer [50 mM Hepes-NaOH (pH 8), 100 mM NaCl, 1 mM EGTA, 0.5% NP40, 2.5 mM MgCl_2_, 1 mM DTT, and 10% glycerol, supplemented with protease and phosphatase inhibitors] was added, and cells were kept on ice for another 15 min. Cells were scraped into tubes and spun down at 4 **°**C for 15 min. Then, 20 μL was taken as input sample and frozen immediately. The rest of the lysates were added to 25 μl washed magnetic streptavidin-Dynabeads (ThermoFisher) and rotated at 4 **°**C for 3–4 h. Beads were washed three times with lysis buffer. After the third wash, 40 μl 2x sample buffer supplemented with β-mercaptoethanol was added to the beads, boiled for 5 min, and frozen at − 20 **°**C. EGFR bands were quantified using ImageJ software and normalized against transferrin receptor. Statistical significance was assessed by one-way ANOVA.

The following are the supplementary data related to this article:

Supplementary figures. Image 2

Supplementary Table 1 Bioinformatic analysis of hits. Supplementary Table 1

Supplementary Table 2 List of plasmids Supplementary Table 2

Supplementary Table 3 List of primers Supplementary Table 3

Supplementary Table 4 List of antibodies Supplementary Table 4

## Figures and Tables

**Table 1:** GO localization and GO function of 19 GFP-Grb2 localization hits

	UniProt	GFP-Grb2 localization	GO localization	GO function
AMPH	P49418	Spots in cytoplasm	Cytoplasm, cytoskeleton, *trans*-Golgi	Regulation of GTPase activity
APBB1IP	Q7Z5R6	Spots in cytoplasm, nucleus	Cytosplasm, cytoskeleton, plasma membrane	Signal transduction
BCL6	P41182	Small spots in nucleus	Nucleus	Regulation of GTPase activity, signaling, transcription
CNK2	Q8WXI2	Spots in cytoplasm	Cytoplasm, Golgi apparatus, membrane	Regulation of signal transduction
DDX17	Q92841	Single spot in cytoplasm	Nucleus	Regulation of transcription, RNA helicase
DIAPH1	O60610	Spots in cytoplasm	Cytoplasm, cytoskeleton, plasma membrane	Actin cytoskeleton organization
DOK3	Q7L591	Spots in cytoplasm	Cytoplasm, plasma membrane	Ras protein signal transduction
ENAH	Q8N8S7	Few spots in nucleus	Cytoplasm, cytoskeleton, focal adhesion	Actin cytoskeleton organization
LZTS2	Q9BRK4	Clustered perinuclear spots	Cytoplasm, centrosome, cytoskeleton	Cell division, Wnt signaling pathway
MBIP	Q9NS73	Few spots in cytoplasm	Cytoplasm, nucleus	Chromatin organization
PHF8	Q9UPP1	Few spots in cytoplasm	Nucleus	Mitotic cell cycle, chromatin organization
PRAM1	Q96QH2	Few spots in nucleus	NA	Integrin-mediated signaling pathway
RIMS2	Q9UQ26	Single spot in cytoplasm	Plasma membrane	cAMP-mediated signaling, regulation of exocytosis
SHKBP1	Q8TBC3	Clustered spots in cytoplasm	NA	Positive regulation of EGFR signaling
SKAP2	O75563	Few spots in cytoplasm	Cytoplasm, plasma membrane	Signal transduction
ST5	P78524	Few spots in cytoplasm	NA	Regulation of GTPase activity
TACC3	Q9Y6A5	Large spots in cytoplasm	Cytoplasm, microtubule, cytoskeleton	Microtubule cytoskeleton organization
TUFT1	Q9NNX1	Spots in cytoplasm, nucleus	Cytoplasm, extracellular region	Intracellular signal transduction
Usp33	Q8TEY7	Few spots in cytoplasm	Cytoplasm, cytoskeleton, Golgi	Protein deubiquitination

NA – not annotated

## Footnotes

† http://kmplot.com/analysis/
