# Supplementary material for: Systematic Identification of Oncogenic EGFR Interaction Partners
Source: J Mol Biol. 2017 Jan 20;429(2):280–94. doi: 10.1016/j.jmb.2016.12.006 (PMC5240790; doi:10.1016/j.jmb.2016.12.006)
Supplement: Supplementary file 1 — Supplementary figures. [file mmc1.pdf]

Supplementary Figure 1.

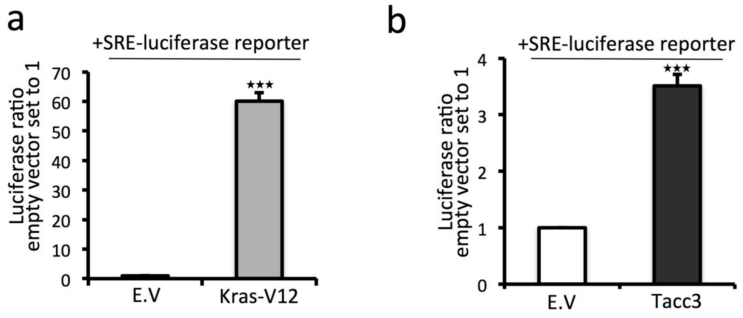

Supplementary Figure 1. Related to Figure 5.

a) HEK293T cells were co-transfected with Kras-V12 or empty vector control and SRE-luciferase reporter. 3 days after transfection, cells were lysed and luciferase activity was measured. Values were normalized to the empty control vector and changes in activity are displayed as fold values. Data are presented as means ± standard deviation (n=3). *Asterisks* show p values: $^{***}p < 0.001$.

b) HEK293T cells were co-transfected with FLAG-tagged Tacc3 or empty vector control and SRE-luciferase reporter. 2 days after transfection, cells were starved overnight and the next day, cells were lysed and luciferase activity was measured. Values were normalized to the empty control vector and changes in activity are displayed as fold values. Data are presented as means ± standard deviation (n=3). *Asterisks* show p values: $^{***}p < 0.001$.

Supplementary Figure 2.

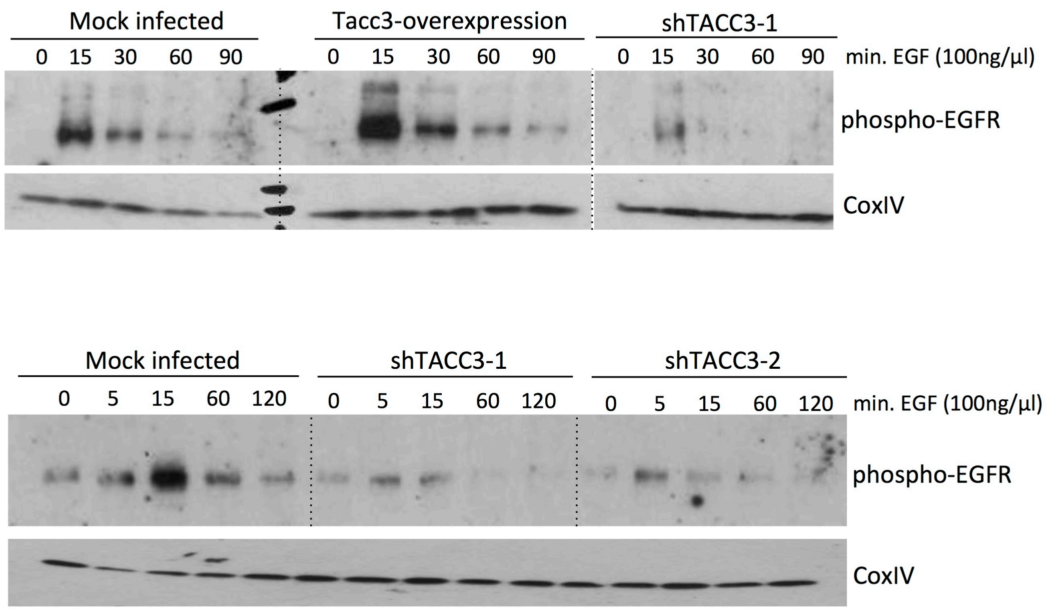

Supplementary Figure 2. Related to Figure 5.

HEK293T cells were infected with indicated lentivirus (upper panel: shGFP control, shTACC3-1 or FLAG-Tacc3; lower panel: shGFP, shTACC3-1 or shTACC3-2) and selected for at least 48 h to obtain stable cells. Cells were then seeded, starved overnight and stimulated with EGF (100 ng/µl) for indicated times. Cells were lysed and protein levels were assessed by Western blot using phosphor-EGFR and CoxIV antibody as loading control.
