# Supplementary material for: Systematic Identification of Oncogenic EGFR Interaction Partners
Source: J Mol Biol. 2017 Jan 20;429(2):280–94. doi: 10.1016/j.jmb.2016.12.006 (PMC5240790; doi:10.1016/j.jmb.2016.12.006)
Supplement: Supplementary Table 2 — List of plasmids [file mmc3.docx]

**Supplementary Table 2.** List of plasmids.

| **Entry Clones** | **backbone** |
| --- | --- |
| EGFR-Wt | EGFR-wildtype in pDONR223 |
| EGFR-L858R | EGFR-L858R in pDONR223 |
| EGFR-T790M/L858R | EGFR-T790M/L858R in pDONR223 |
| EGFR-ex19del | EGFR-ex19del in pDONR223 |
| Amph | pDONR223 (hORFeome v5.1) |
| Tacc3 | pDONR223 (hORFeome v5.1) |
| Usp33 | pDONR223 (hORFeome v5.1) |
| Cnk2 | pDONR223 (hORFeome v5.1) |
| Mbip | pDONR223 (hORFeome v5.1) |
| Diaph1 | pDONR223 (hORFeome v5.1) |
| Dok3 | pDONR223 (hORFeome v5.1) |
| Lzts2 | pDONR223 (hORFeome v5.1) |
| Skap2 | pDONR223 (hORFeome v5.1) |
| Ddx17 | pDONR223 (hORFeome v5.1) |
| Rim2 | pDONR223 (hORFeome v5.1) |
| Apbb1ip | pDONR223 (hORFeome v5.1) |
| Shkbp1 | pDONR223 (hORFeome v5.1) |
| Phf8 | pDONR223 (hORFeome v5.1) |
| St5 | pDONR223 (hORFeome v5.1) |
| Tuft1 | pDONR223 (hORFeome v5.1) |
| Enah | pDONR223 (hORFeome v5.1) |
| Pram | pDONR223 (hORFeome v5.1) |
| Bcl6 | pDONR223 (hORFeome v5.1) |
|  |  |
| **Expression vectors** |  |
| GFP-Grb2 | pEAK14-GFP-Grb2 |
| EGFR-Wt-bait | pCMV-Gateway-linker-GAL4-mNFkB-V5 [12] |
| EGFR-L858R-bait | pCMV-Gateway-linker-GAL4-mNFkB-V5 [12] |
| EGFR-T790M/L858R-bait | pCMV-Gateway-linker-GAL4-mNFkB-V5 [12] |
| EGFR-ex19del-bait | pCMV-Gateway-linker-GAL4-mNFkB-V5 [12] |
| 19 preys (see above) | pCMV-hNubI-tripleFLAG-linker-Gateway [12] |
| lentiviral 3xFLAG-Tacc3 | pLV-CMV-tripleFLAG-linker-Gateway-PuroR [12] |
| EGFR-Wt-GFP | pCMV-Gateway-GFP (C-terminal) |
| EGFR-L858R-GFP | pCMV-Gateway-GFP (C-terminal) |
| EGFR-T790M/L858R-GFP | pCMV-Gateway-GFP (C-terminal) |
| EGFR-ex19del-GFP | pCMV-Gateway-GFP (C-terminal) |
|  |  |
| **lentiviral shRNA-constructs** |  |
| shTACC3 | pLKO.1 TACC3 shRNA (NM_006342.1-300s21c1) |
| shGFP | pLKO.1 GFP shRNA |
