# Supplementary material for: Systematic Identification of Oncogenic EGFR Interaction Partners
Source: J Mol Biol. 2017 Jan 20;429(2):280–94. doi: 10.1016/j.jmb.2016.12.006 (PMC5240790; doi:10.1016/j.jmb.2016.12.006)
Supplement: Supplementary Table 3 — List of primers [file mmc4.docx]

**Supplementary Table 3.** List of primers.

| **mutagenesis primers** |  |
| --- | --- |
| EGFR-L858R_F | CACAGATTTTGGGCgGGCCAAACTGCTGGG |
| EGFR-L858R_R | CCCAGCAGTTTGGCCcGCCCAAAATCTGTG |
| EGFR-ex19del_F | GTTAAAATTCCCGTCGCTATCAAGACATCTCCGAAAGCCAACAAGG |
| EGFR-ex19del_R | CCTTGTTGGCTTTCGGAGATGTCTTGATAGCGACGGGAATTTTAAC |
| EGFR-T790M_F | GTGCAGCTCATCATGCAGCTCATGCC |
| EGFR-T790M_R | GGCATGAGCTGCATGATGAGCTGCAC |
|  |  |
| **entry clone generation** |  |
| EGFR_F | GGGGACAAGTTTGTACAAAAAAGCAGGCTTA gccacc atgcgaccctccgggacgg |
| EGFR-R | GGGGACCACTTTGTACAAGAAAGCTGGGTA tgctccaataaattcactgctttg |
| Shc1_F | GGGGACAAGTTTGTACAAAAAAGCAGGCTTA atgaacaagctgagtggaggcg |
| Shc1_R | GGGGACCACTTTGTACAAGAAAGCTGGGTA tcacagtttccgctccacagg |
| Tacc3_F | GGGGACAAGTTTGTACAAAAAAGCAGGCTTA atgagtctgcaggtcttaaacg |
| Tacc3_R | GGGGACCACTTTGTACAAGAAAGCTGGGTA tcagatcttctccatcttggag |
