# Supplementary material for: Systematic Identification of Oncogenic EGFR Interaction Partners
Source: J Mol Biol. 2017 Jan 20;429(2):280–94. doi: 10.1016/j.jmb.2016.12.006 (PMC5240790; doi:10.1016/j.jmb.2016.12.006)
Supplement: Supplementary Table 4 — List of antibodies [file mmc5.docx]

**Supplementary Table 4**. List of antibodies.

| anti-ERK1/2 | Rabbit, p44/42 MAPK 45955, Cell Signaling | 1:1000 |
| --- | --- | --- |
| anti-phospho-ERK1/2 | Rabbit, p44/42 MAPK (T202/Y204) 4370S, Cell Signaling | 1:1000 |
| anti-GFP | Mouse, JL-8, Living Colors | 1:1000 |
| anti-EGFR | Rabbit, 1005, sc-03, Santa Cruz | 1:750 |
| anti-phospho-EGFR | Rabbit, pTyr 1068, LS-C6640, Life Span, | 1:1000 |
| anti-FLAG | Mouse, M2, F3165 Sigma | 1:1000 |
| anti-CoxIV | Rabbit, 3E11, Cell Signaling 4850S | 1:1000 |
| anti-Tacc3 | Rabbit, H-300, Santa Cruz sc-22773 | 1:750 |
| anti-transferrin receptor | Rabbit, ab84036, abcam | 1:1000 |
| anti-AKT | Rabbit, 9272, Cell Signaling | 1:1000 |
| anti-phospho-AKT | Rabbit, Ser473, Cell Signaling 4058 | 1:1000 |
| anti-mouse IgG | HRP-linked (goat), Santa Cruz sc-2005 | 1:10,000 |
| anti-rabbit IgG | HRP-linked (goat), Santa Cruz sc-2004 | 1:10,000 |
